# Randomized pharmacokinetic and drug–drug interaction studies of ceftazidime, avibactam, and metronidazole in healthy subjects

**DOI:** 10.1002/prp2.172

**Published:** 2015-08-25

**Authors:** Shampa Das, Jianguo Li, Jon Armstrong, Maria Learoyd, Timi Edeki

**Affiliations:** 1 AstraZenecaMacclesfield, United Kingdom; 2 AstraZenecaWaltham, Massachusetts; 3 AstraZenecaWilmington, Delaware

**Keywords:** Avibactam, ceftazidime, drug–drug interaction, infectious disease, pharmacokinetics

## Abstract

We assessed pharmacokinetic and safety profiles of ceftazidime–avibactam administered ± metronidazole, and whether drug–drug interactions exist between ceftazidime and avibactam, or ceftazidime-avibactam and metronidazole. The first study (NCT01430910) involved two cohorts of healthy subjects. Cohort 1 received ceftazidime–avibactam (2000–500 mg) as a single infusion or as multiple intravenous infusions over 11 days to evaluate ceftazidime–avibactam pharmacokinetics. Cohort 2 received ceftazidime, avibactam, or ceftazidime–avibactam over 4 days to assess drug–drug interaction between ceftazidime and avibactam. The second study (NCT01534247) assessed interaction between ceftazidime–avibactam and metronidazole in subjects receiving ceftazidime–avibactam (2000–500 mg), metronidazole (500 mg), or metronidazole followed by ceftazidime–avibactam over 4 days. In all studies, subjects received a single-dose on the first and final days, and multiple-doses every 8 h on intervening days. Concentration-time profiles for ceftazidime and avibactam administered as single- or multiple-doses separately or together with/without metronidazole were similar. There was no evidence of time-dependent pharmacokinetics or accumulation. In both interaction studies, 90% confidence intervals for geometric least squares mean ratios of area under the curve and maximum plasma concentrations for each drug were within the predefined interval (80–125%) indicating no drug–drug interaction between ceftazidime and avibactam, or ceftazidime–avibactam and metronidazole. There were no safety concerns. In conclusion, pharmacokinetic parameters and safety of ceftazidime, avibactam, and metronidazole were similar after single and multiple doses with no observed drug–drug interaction between ceftazidime and avibactam, or ceftazidime–avibactam and metronidazole.

## Introduction

Treatment options for infections caused by Gram-negative pathogens, especially multidrug-resistant strains and those producing extended-spectrum *β*-lactamases, are currently very limited (Hirsch and Tam 2010; Peleg and Hooper 2010; Kanj and Kanafani 2011; Boucher et al. 2013). Avibactam is a novel non-*β*-lactam *β*-lactamase inhibitor that inhibits Ambler class A *β*-lactamases, including *Klebsiella pneumoniae* carbapenemases, Ambler class C and some Ambler class D *β*-lactamases (Stachyra et al. 2009). Avibactam can restore the in vitro activity of *β-*lactams, including ceftazidime, aztreonam, and ceftaroline, the active component of the prodrug ceftaroline fosamil, against extended spectrum *β*-lactamase-producing pathogens (Lagace-Wiens et al. 2011; Livermore et al. 2011).

Ceftazidime–avibactam has been shown to be effective and generally well tolerated in phase II clinical trials of patients with complicated intra-abdominal infections (cIAI) or complicated urinary tract infections (cUTI) (Vazquez et al. 2012; Lucasti et al. 2013). These promising results have led to further evaluation of ceftazidime 2000 mg in combination with avibactam 500 mg in phase III trials of patients with cIAI (NCT01726023, NCT01499290, and NCT01500239), cUTI (NCT01595438, and NCT01599806), nosocomial pneumonia (NCT01808092) and infections with ceftazidime-resistant Gram-negative pathogens (NCT01644643). Given that cIAI can be polymicrobial in nature, it is important to provide antimicrobial therapy that has activity against anaerobic as well as aerobic Gram-negative pathogens. Therefore, in trials of patients with cIAI, metronidazole is coadministered with ceftazidime–avibactam to provide anaerobic coverage.

Here, we report data from two phase I pharmacokinetic studies in healthy subjects, the main objectives of which were to determine the pharmacokinetics of ceftazidime and avibactam following intravenous (IV) infusions, and to determine if there were drug–drug interactions between ceftazidime and avibactam, or between ceftazidime–avibactam and metronidazole, by comparing the pharmacokinetics of the compounds when they were administered separately or in combination. Safety and tolerability were also evaluated.

The doses, duration of the infusions and frequency of dosing used in these phase I studies were chosen to replicate those being evaluated in phase III clinical trials, namely ceftazidime 2000 mg in combination with avibactam 500 mg given as a 2-h IV infusion every 8 h (q8 h), with coadministration of metronidazole 500 mg given as a 1-h IV infusion q8 h in cases of cIAI (a schedule consistent with the approved dosing regimen for metronidazole) (Winthrop Pharmaceuticals UK Limited 2013). Where used, metronidazole infusions were given before the ceftazidime–avibactam infusions.

## Materials and Methods

Two separate phase I, open-label clinical trials were conducted in healthy subjects. The first (NCT01430910; sponsor protocol number: D4280C00011) was the “CAZ and AVI drug–drug interaction study” and was conducted in two parts. The “CAZ-AVI PK study” was the first part, which evaluated the pharmacokinetics of ceftazidime–avibactam after single and multiple doses, reflecting clinical administration of the antibiotic combination that typically occurs over 5–10 days. The “CAZ and AVI interaction study” was the second part, which assessed whether there was any drug–drug interaction between ceftazidime and avibactam. The second clinical trial (NCT01534247; sponsor protocol number: D4280C00012) was the “CAZ-AVI and MTZ interaction study” and assessed whether there was drug–drug interaction between ceftazidime–avibactam and metronidazole.

The CAZ-AVI PK part of the first trial was conducted at Quintiles Drug Research Unit, Guy's Hospital, London, UK, and the CAZ and AVI interaction part of this first trial was conducted at Hammersmith Medicines Research, London, UK, from October 2011 to October 2012. The second trial, evaluating ceftazidime–avibactam and metronidazole interaction, was conducted at Quintiles Phase I Services, Overland Park, KS, from February 2012 to July 2012. The protocols for each study were approved by the appropriate Institutional Review Board (Edinburgh, UK and Overland Park, KS, respectively). All studies were performed in accordance with the ethical principles of the Declaration of Helsinki and in compliance with Good Clinical Practice. All participants provided written informed consent before the respective studies started.

### Subjects

In all studies, healthy adult male or female subjects aged 18–50 years inclusive and with body mass index 19–30 kg/m^2^ were eligible for inclusion. Females had to be of nonchildbearing potential. Exclusion criteria included history of any clinically significant disease or disorder which rendered the subject at risk from participation in the study, or could influence the results or the subjects ability to participate; history or presence of gastrointestinal, hepatic, or renal disease, or any other condition known to interfere with the pharmacokinetics of drugs; history of any hypersensitivity to drugs with a similar chemical structure or class to ceftazidime, avibactam, metronidazole, and/or excipients; the presence, as judged by the investigator, of any clinically significant abnormalities in the subjects’ vital signs (systolic blood pressure >140 mmHg; diastolic blood pressure >90 mmHg; or heart rate <40 or >85 beats per min after 10 min supine rest) or electrocardiogram (ECG); use of any prescribed or nonprescribed medications 2 weeks prior to the first administration of the study drug (or longer if medication has a long half-life), including antacids, herbal remedies, vitamins, minerals, and analgesics, with the exception of occasional doses of paracetamol/acetaminophen; current or ex-smokers who have used any nicotine-containing product within the 3 months; plasma donation within 1 month of screening or blood donation or blood loss during the previous 3 months prior to screening; intake of grapefruit or Seville oranges or their products within 7 days of the first administration of the study treatment.

### Study designs

Two separate cohorts of healthy subjects participated in the two parts of the CAZ and AVI drug–drug interaction study: the CAZ-AVI PK study (*n *=* *16) and the CAZ and AVI interaction study (*n *=* *27). The primary objective of the CAZ−AVI PK part was to investigate the pharmacokinetic parameters and time-dependence of ceftazidime 2000 mg plus avibactam 500 mg following a single dose on Days 1 and 11 and multiple doses (q8 h) on Days 2 to 10, thereby determining when the steady state may be achieved. Ceftazidime–avibactam was administered over a 10-day period to ensure that adequate drug concentrations were maintained, reflecting clinical practice. Each dose was given as a 2-h IV infusion.

The CAZ and AVI interaction part of the CAZ and AVI drug–drug interaction study had a 3-way crossover design, and the primary objective was to investigate the effect on pharmacokinetics of coadministering ceftazidime and avibactam compared with separate administration of the individual components, to determine if there was a drug–drug interaction between the two. Each subject was to receive each of the following three treatments in randomized order: ceftazidime 2000 mg, avibactam 500 mg, or ceftazidime 2000 mg plus avibactam 500 mg. Each treatment was administered as a 2-h IV infusion, given once on Days 1 and 4 and q8 h on Days 2 and 3. There was a washout period of at least 48 h between each treatment period to avoid carryover.

The CAZ-AVI and MTZ interaction study also had a 3-way crossover design. The primary objective was to determine whether there was any drug–drug interaction between ceftazidime–avibactam and metronidazole by comparing the pharmacokinetics of ceftazidime–avibactam and metronidazole when the drugs were administered in combination (ceftazidime–avibactam plus metronidazole), or as separate administrations (either ceftazidime–avibactam alone or metronidazole alone). Subjects (*n *=* *28) each received all of the following three treatments in randomized order: 2-h infusion of ceftazidime 2000 mg-avibactam 500 mg; 1−h infusion of metronidazole 500 mg; 1-h infusion of metronidazole 500 mg followed by a 2-h infusion of ceftazidime 2000 mg-avibactam 500 mg. The IV line was flushed with saline solution between administration of metronidazole and ceftazidime–avibactam. Each component of each treatment was administered once in the morning of Day 1, q8 h on Days 2, and 3, and once again on Day 4, hence each subject received each component a total of 8 times during the treatment period. Each treatment period was separated with a wash-out period of at least 48 h.

### Pharmacokinetic analysis

In the CAZ-AVI PK part of the CAZ and AVI drug–drug interaction study, blood samples were collected on Day 1 (at 0 h [predose], 0.5, 1, 1.5, 2, 2.25, 2.5, 2.75, 3, 3.5, 4, 6, 8, 12 and 24 h [Day 2 predose]) postdose, Day 4 (at 0 h [predose], at 1, 2, 3, 4, and 6 h, and at 8 h [after the first dose and prior to the second dose]), and Day 11 (same schedule as for Day 1). Urine samples were collected on Day 1 and Day 11 during the following intervals: 0–2 h, 2–4 h, 4–8 h, 8–12 h, and 12–24 h. In the CAZ and AVI interaction part of the CAZ and AVI drug–drug interaction study, blood and urine samples were collected on Days 1 and 4 of each treatment period, according to the same schedules as for Day 1 in the CAZ-AVI PK part.

In the CAZ-AVI and MTZ interaction study, blood samples were collected at the following times for each treatment (all times being relative to the start of the first infusion):

1. For the ceftazidime–avibactam group, blood samples were collected on Day 1 and Day at 0 h (predose), 0.5, 1, 1.5, 2 (end of ceftazidime–avibactam infusion), 2.25, 2.5, 2.75, 3, 4, 5, 7, 11, and 23 h (prior to the second dose for Day 1)
2. For the metronidazole group, blood samples were collected on Day 1 and Day 4 at 0 h (predose), 0.5, 1 (end of metronidazole infusion), 1.25, 1.5, 2, 3, 4, 6, 8, 12, and 24 h (prior to the second dose for Day 1)
3. For the ceftazidime–avibactam plus metronidazole group, blood samples were collected on Day 1 and Day 4 at 0 h (predose), 0.5, 1 (end of metronidazole infusion, prior to ceftazidime–avibactam infusion), 1.25, 1.5, 2, 2.5, 3 (end of ceftazidime–avibactam infusion), 3.25, 3.5, 3.75, 4, 5, 6, 8, 12, and 24 h (prior to the second dose for Day 1).

Urine samples were collected on Days 1 and 4 of each treatment period, according to the same schedules as for Day 1 in the CAZ-AVI PK part of the CAZ and AVI drug–drug interaction study.

In all studies, drug concentrations in plasma and urine were analyzed by Quotient Bioresearch Ltd (Fordham, UK). Drug concentrations were determined by validated bioanalytical assays involving ultraperformance liquid chromatography followed by pneumatic-assisted electrospray (TurboIonSpray∼, Applied BioSystems/MDS Sciex Concord, ON, Canada) tandem mass-spectrometry (Sillén et al. 2015). Sodium fluoride/potassium oxalate was used as an anticoagulant for the human plasma samples used for pharmacokinetic analysis.

In both parts of the CAZ and AVI drug–drug interaction study, plasma concentrations of ceftazidime (Sigma-Aldrich, St Louis, MO) were determined as previously described (Das et al. 2014). Precision (% coefficient of variation [CV%]) and accuracy (% relative error [RE%]) were ≤7.3% and 1.5–3.7%, respectively (high range), and ≤4.9% and 0.0–0.3%, respectively (low range). In the CAZ-AVI and MTZ interaction study, validated concentration ranges of 44.6–892 ng/mL (low range) and 446–89,200 ng/mL (high range) were used and the precision (CV%) and accuracy (RE%) were ≤6.8% and −1.0–0.4%, respectively (high range), and ≤4.1% and −0.8–0.3%, respectively (low range).

Avibactam (AstraZeneca, Macclesfield, UK) was solid-phase extracted from plasma samples using Oasis® WAX plates (Waters, Milford, MA), and concentrations were determined over two validated calibration ranges (10–1000 ng/mL and 500–50,000 ng/mL) with a validated dilution of 10-fold with human plasma and using ^13^C_5_/^15^N avibactam as internal standard. In both parts of the CAZ and AVI drug–drug interaction study, precision (CV%) and accuracy (RE%) were ≤7.1% and −0.5–0.0%, respectively (high range), and ≤6.3% and 0.0–4.0%, respectively (low range). In the CAZ-AVI and MTZ interaction study, precision (CV%) and accuracy (RE%) were ≤7.0% and −1.3–1.0%, respectively (high range), and ≤5.4% and 2.0–5.7%, respectively (low range).

For the determination of metronidazole concentration, 50 *μ*L plasma samples were extracted using an Isolute® SLE+ plate (Biotage AB, Uppsala, Sweden) and subjected to ultraperformance liquid chromatography and pneumatic assisted electrospray (TurboIonSpray∼) tandem mass spectrometry. Metronidazole concentrations were determined over a validated calibration range of 40.0–8000 ng/mL with a validated dilution of 10-fold with human plasma and with metronidazole-d4 (Toronto Research Chemicals, North York, ON, Canada) as the internal standard. Precision (CV%) and accuracy (RE%) were ≤6.0% and −3.9 to −1.7%, respectively. The lower limit of quantification (LLOQ) was previously established as 40.0 ng/mL during the method validation performed prior to the plasma analysis, using a 50 *μ*L sample volume.

Urine samples were diluted with 10 mmol/L ammonium formate (aqueous) (pH 3) for analysis of ceftazidime and with acetonitrile: 100 mmol/L ammonium formate (aqueous) pH 9 (95:5, v/v) for analysis of avibactam. Ceftazidime concentrations in urine were determined using validated calibration ranges of 437–268,000 ng/mL with a validated 10-fold dilution with human urine. A validated calibration range of 500–300,000 ng/mL with a validated 50-fold dilution with human urine was used for determination of avibactam in human urine. Data collection and peak integration were performed using Analyst® software version 1.5.1 and 1.5.2 (Applied Biosystems/MDS Sciex, Foster City, CA). Standard regression and quantitation were performed using Watson LIMS version 7.2 (Thermo Scientific, Philadelphia, PA).

For all studies, the following pharmacokinetic parameters were calculated for ceftazidime, avibactam, and metronidazole (where applicable): the area under the plasma concentration-time curve from time zero to infinity (AUC), the AUC during the dosing interval (AUC_[0-*τ*]_), the accumulation ratio for AUC_(0-*τ*)_ (AUC_[0-*τ*]_ at steady-state/AUC_[0-*τ*]_ on Day 1), the linearity index determined as the ratio of steady-state AUC_(0-*τ*)_ to Day 1 AUC, and the maximum plasma concentration (*C*_max_). Data are presented as the geometric mean and the geometric CV% (calculated as 100 · √[exp (s^2^) − 1], where s is the standard deviation [SD] of the log scale data). AUC_(0-*τ*)_ was calculated by linear up/log down trapezoidal summation and AUC was calculated by linear up/log down trapezoidal summation and extrapolated to infinity by addition of the last quantifiable concentration (*C*_last_) divided by the elimination rate constant (*λ*_z_). If the extrapolated area (*C*_last_/*λ*_z_) was greater than 20%, AUC and related parameters were not calculated. Also calculated were the median (range) time to maximum plasma concentration; and the arithmetic mean (SD) plasma and renal clearance (CL and CL_R_, respectively) and terminal elimination half-life. Calculations were completed using actual sampling times, by noncompartmental methods using WinNonlin® Professional Version 5.2 or higher (Pharsight Corp., Mountain View, CA) or SAS® Version 9.1 or higher (SAS Institute, Inc., Cary, NC) at Quintiles (Overland Park, KS).

### Safety

Safety was evaluated in all studies by assessment of adverse events (AEs), clinical laboratory parameters, vital signs (blood pressure, pulse rate), digital and 12-lead ECG on Days –1, 4, 7, and before discharge from the study center, and physical examinations. AEs were summarized for each study using Preferred Term and System Organ Class according to the Medical Dictionary for Regulatory Activities (MedDRA version 13.1; MedDRA MSSO, Chantilly, VA).

### Statistical analysis

For the drug–drug interaction parts of both the CAZ and AVI drug–drug interaction study and the CAZ-AVI and MTZ interaction study, it was estimated that 24 evaluable subjects (in each study) would provide approximately 90% power to confirm that a combined treatment had no effect on the pharmacokinetics of each of the individual treatment components. The power calculation was based on equivalence testing using standard equivalence limits of (0.8, 1.25) (Food and Drug Administration 2012), to compare the *C*_max_ of each compound after separate and combined administration of ceftazidime and avibactam in the CAZ and AVI interaction part of the CAZ and AVI drug–drug interaction study, and ceftazidime–avibactam and metronidazole in the CAZ-AVI and MTZ interaction study.

The within-subject SD of log (avibactam *C*_max_) and log (ceftazidime *C*_max_) were estimated as 0.2088 and 0.2171, respectively, based on previous experience with ceftazidime–avibactam; the within-subject variability of log (metronidazole *C*_max_) was assumed to be no greater in magnitude. The estimates of within-subject variability for the AUC were lower than for *C*_max_; therefore, this sample size also provided sufficient power for comparisons involving AUC based on equivalence testing, using the standard equivalence limits of (0.8, 1.25).

Geometric least-squares (LS) mean ratios with 90% confidence intervals (CI) for the AUC (Day 1 only), AUC_(0-*τ*)_ (Day 4 only), and *C*_max_ (on both Days 1 and 4) were calculated.

Analysis was performed by day (Day 1 and Day 4) with a linear mixed-effects model, using the log AUC, AUC_(0-*τ*)_, and *C*_max_ as response variables, sequence, period, and treatment as fixed effects, and the healthy volunteer nested within sequence as random effect. If the 90% CI for the geometric LS mean ratios were within the interval of 80–125% for no interaction effect (applying an equivalence approach as per the US Food and Drug Administration guidance for drug–drug interaction studies) (Food and Drug Administration 2012), then it could be concluded that ceftazidime did not affect exposure to avibactam (or *vice versa*) in the CAZ and AVI interaction part of the CAZ and AVI drug–drug interaction study, or that metronidazole did not affect exposure to ceftazidime–avibactam (or *vice versa*) in the CAZ-AVI and MTZ interaction study. An exploratory evaluation of achievement of steady-state was performed graphically in the CAZ-AVI PK part of the CAZ and AVI drug–drug interaction study. All statistical analyses were performed using SAS® Version 9.2.

## Results

Subject baseline characteristics are shown in Table1. Sixteen subjects participated in and completed the CAZ-AVI PK part of the CAZ and AVI drug–drug interaction study. All subjects were included in the safety and pharmacokinetic analyses.

**Table 1:** Patient baseline characteristics (randomized population)

	CAZ and AVI drug–drug interaction study		
	CAZ-AVI PK (*n* = 16)	CAZ and AVI interaction (all groups combined) (*n* = 27)	CAZ-AVI and MTZ interaction study (*n* = 28)
Age, years, mean (SD)	32 (8)	32 (9)	31 (7)
Males, %	100	100	100
Race, *n* (%)			
Asian	4 (25.0)	6 (22.2)	0 (0.0)
Black or African-American	0 (0.0)	4 (14.8)	6 (21.4)
White	12 (75.0)	16 (59.3)	22 (78.6)
Other	0 (0.0)	1 (3.7)	0 (0.0)
Weight, kg, mean (SD)	77.2 (8.8)	77.6 (11.0)	78.4 (12.2)
BMI, kg/m^2^, mean (SD)	24.2 (2.6)	24.3 (2.3)	24.6 (3.4)

AVI, avibactam; BMI, body mass index; CAZ, ceftazidime; MTZ, metronidazole; PK, pharmacokinetics; SD, standard deviation.

In the CAZ and AVI interaction part of the CAZ and AVI drug–drug interaction study, 27 subjects were randomized and all completed the study. One subject in the ceftazidime–avibactam group of this study had an abnormal ceftazidime pharmacokinetic profile with peak ceftazidime concentration observed at predose, and very low concentrations observed on Day 4 compared with other subjects in the group. Consequently, that subject's data were excluded from the Day 4 ceftazidime pharmacokinetic and statistical analysis. Pharmacokinetic parameters for avibactam on Day 4 in the same subject appeared similar to those of other subjects in the same group and were therefore included in the analysis. The subject was included in the safety analysis.

In the CAZ-AVI and MTZ interaction study, 28 subjects were randomized, of whom 27 received all 3 study treatments and completed the study. One subject withdrew consent prior to commencing metronidazole in period 3 after multiple unsuccessful attempts to start the drawing of blood from his IV catheter. Furthermore, one subject had a metronidazole infusion 20 min longer than planned on Day 4 during the ceftazidime–avibactam plus metronidazole treatment; therefore, the metronidazole pharmacokinetic data for this subject were excluded from the Day 4 analysis. All subjects randomized were included in the safety analyses.

### Pharmacokinetics of ceftazidime–avibactam following single and multiple doses

**Figure 1:**
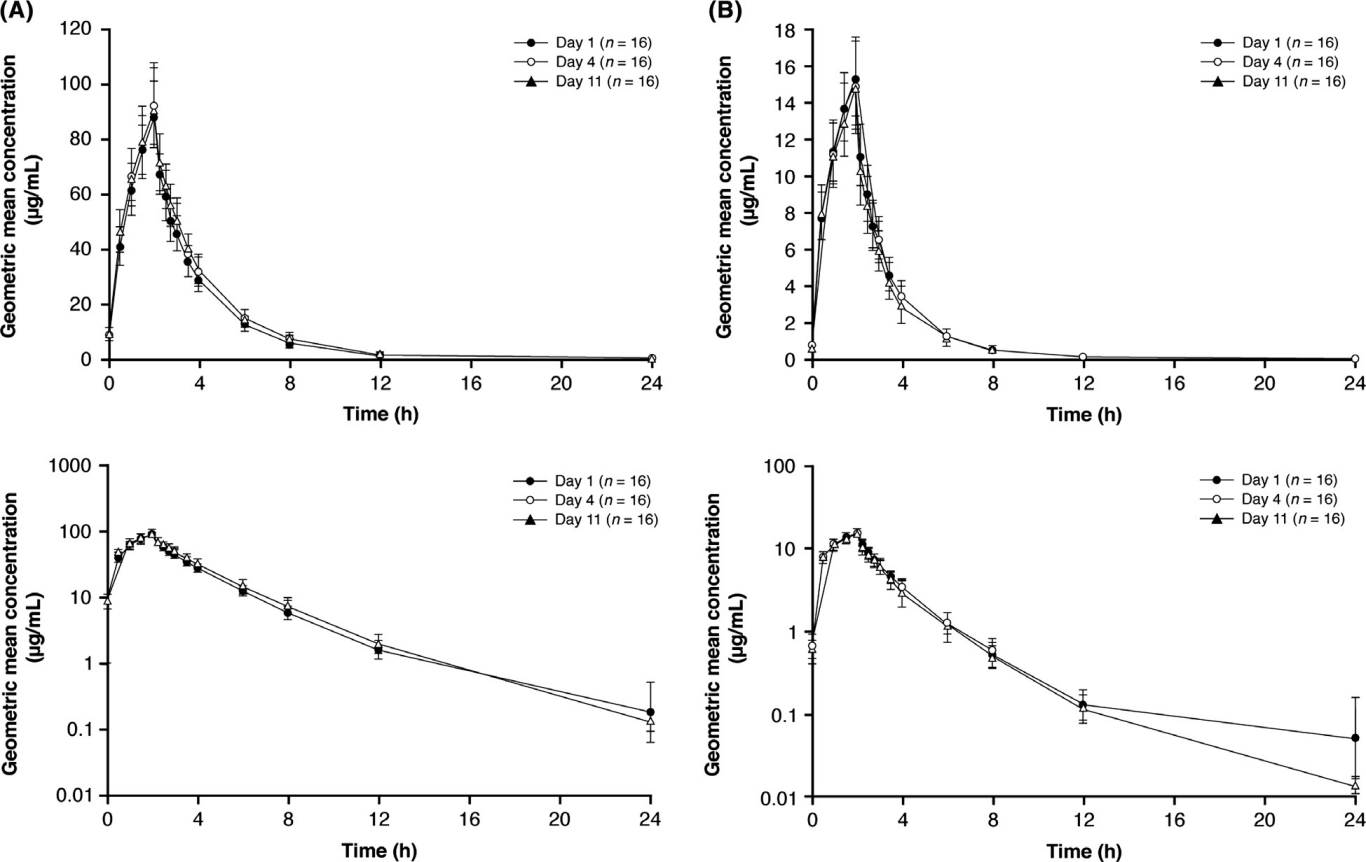
Geometric mean (±SD) plasma concentration-time profiles of (A) ceftazidime and (B) avibactam following single and multiple doses in the CAZ-AVI PK part of the CAZ and AVI drug–drug interaction study. Linear (top) and semilog scales (bottom) are shown. AVI, avibactam; CAZ, ceftazidime; SD, standard deviation.

In the CAZ-AVI PK part of the CAZ and AVI drug–drug interaction study, mean plasma concentration-time profiles (Fig.1) and pharmacokinetic parameters (Table2) of ceftazidime and avibactam were similar following administration of a single dose on Day 1, after multiple dosing (q8 h) on Day 4, and after the single dose on Day 11 (following completion of the multiple-dose treatment period on Days 2 to‒10) (Table2, Fig.1). These results indicated that steady state had been reached prior to 3 days of multiple dosing. Geometric mean accumulation ratios (Day 4/Day 1 and Day 11/Day 1) for AUC_(0-*τ*)_ and *C*_max_ were all close to 1 (Table2), indicating that no accumulation of ceftazidime or avibactam was observed after multiple dosing. Moreover, the geometric mean linearity indices were approximately 1 for ceftazidime and avibactam (Table2), indicating that no time-dependent pharmacokinetics were observed. The arithmetic mean CL and CL_R_ on Day 1 for avibactam were approximately similar (12 and 14 L/h, respectively), showing 100% renal clearance of avibactam in healthy volunteers.

**Table 2:** Summary of key pharmacokinetic parameters: CAZ-AVI PK part of the CAZ and AVI drug–drug interaction study

	Ceftazidime (*n* = 16)			Avibactam (*n* = 16)		
Parameter	Day 1	Day 4	Day 11	Day 1	Day 4	Day 11
AUC (*μ*g × h/mL)	289.0^1^(15.4)	N/A	N/A	42.1^2^(16.0)	N/A	N/A
AUC_(0-*τ*)_ (*μ*g × h/mL)	265.0 (14.4)	294.0 (15.7)	291.0 (15.2)	40.0 (16.1)	39.9 (17.5)	38.2 (18.9)
*C*_max_ (*μ*g/mL)	88.1 (14.0)	92.0 (16.4)	90.4 (15.7)	15.2 (14.1)	14.8 (15.5)	14.6 (17.0)
*t*_max_ (h)	2.0 (2.00–2.02)	2.0 (2.00–2.02)	2.0 (1.50–2.02)	2.0 (2.00–2.02)	2.0 (2.00–2.02)	2.0 (2.00–2.02)
*t*_½_ (h)	3.5^1^(1.3)	1.9 (0.2)	2.8 (0.2)	2.3^2^(0.8)	1.6 (0.1)	2.8 (0.6)
CL	7.0^1^(1.1)	6.9 (1.1)	6.9 (1.0)	12.0^2^(1.8)	12.7 (2.2)	13.3 (2.4)
CL_R_	7.1 (1.4)	N/A	7.1 (1.3)	14.4 (9.4)	N/A	14.1 (3.4)
RAUC_(0-*τ*)_	N/A	1.1 (5.4)	1.1 (5.3)	N/A	1.0 (6.7)	1.0 (11.0)
Linearity index	N/A	1.0 (5.2)	1.0 (5.4)	N/A	1.0 (8.7)^2^	0.9 (11.0)^2^

AUC, area under the plasma concentration-time curve from time zero extrapolated to infinity; AUC_(0-*τ*)_, AUC during the dosing interval; CL, plasma clearance; CL_R_, renal clearance; *C*_max_, maximum plasma concentration; CV, geometric coefficient of variation; *λ*_z_, individual estimate of the terminal elimination rate constant; linearity index, the ratio of steady state AUC_(0-*τ*)_ on Day 4 and Day 11 to Day 1 AUC; *n*, number of subjects; N/A, not applicable; PK, pharmacokinetic; q8 h, every 8 h; RAUC_(0-*τ*)_, accumulation for the ratio of AUC_(0-*τ*)_; Rsq, coefficient of determination for calculation of *λ*_z_; SD, standard deviation; *t*_½_, terminal elimination half-life; *t*_max_, time to maximum plasma concentration. Treatment: ceftazidime 2000 mg and avibactam 500 mg administered intravenously over 2 h, as a single dose on Days 1 and 11 and q8 h on Days 2–10. Data for all parameters are presented as geometric mean (CV%), except for *t*_max_ which are presented as median (range) and *t*_½_, CL and CL_R_ which are presented as arithmetic mean (SD).

1 *n *=* *15, AUC, *t*_½_, CL, and linearity index values not reported for one volunteer on Day 1 as Rsq was less than 0.8 for *λ*_z_ estimation.

2 *n *=* *13, AUC, *t*_½_, CL, and linearity index values not reported for three volunteers on Day 1 as Rsq was less than 0.8 for *λ*_z_ estimation.

**Figure 2:**
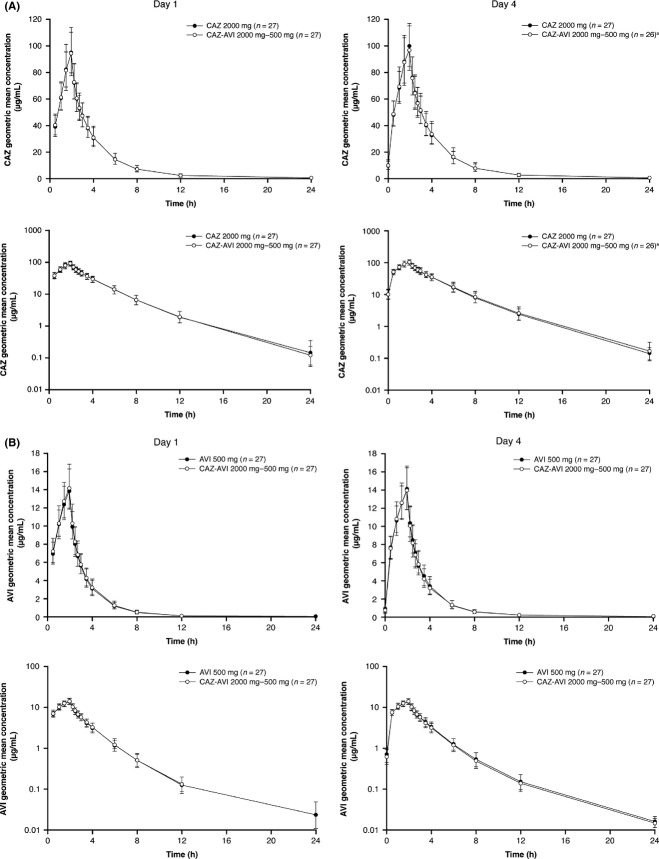
Geometric mean (±SD) plasma concentration-time profiles in the CAZ and AVI interaction part of the CAZ and AVI drug–drug interaction study for (A) ceftazidime 2000 mg when administered singly and in combination with avibactam 500 mg, on linear (top) and semilog scales (bottom), and (B) avibactam 500 mg when administered singly and in combination with ceftazidime 2000 mg, on linear (top) and semilog scales (bottom). AVI, avibactam; CAZ, ceftazidime; SD, standard deviation. ^*^Compared with other subjects in the ceftazidime–avibactam group, one subject had very low concentrations of CAZ on Day 4, and the data were excluded from the Day 4 analysis. Pharmacokinetic parameters for avibactam on Day 4 in the same subject appeared similar to those in other subjects in the same group and were therefore included in the analysis.

**Figure 3:**
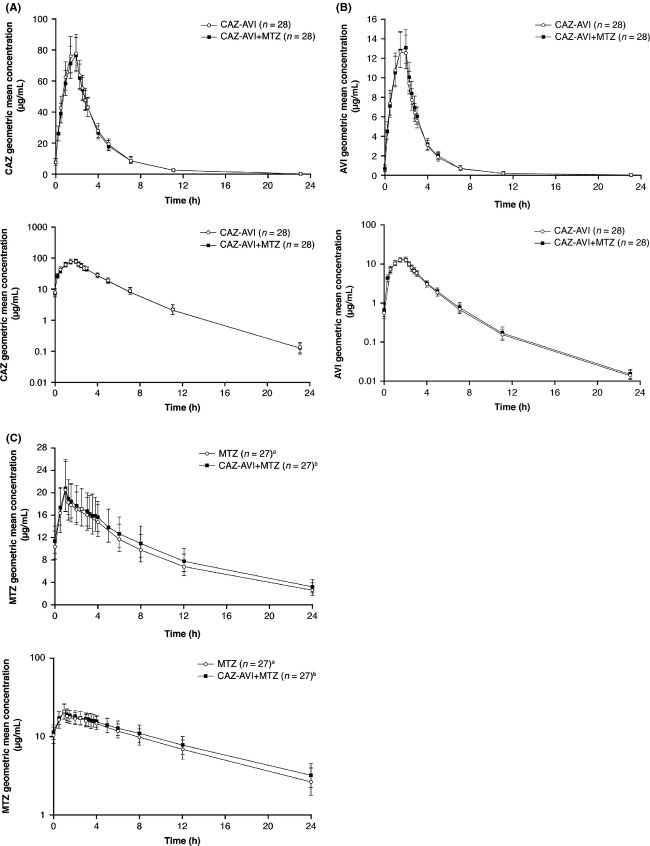
Geometric mean (SD) plasma concentration-time profiles on Day 4 of the CAZ-AVI and MTZ interaction study for ceftazidime (A) and avibactam (B) when administered as ceftazidime 2000 mg-avibactam 500 mg or ceftazidime 2000 mg-avibactam 500 mg plus metronidazole 500 mg, and for metronidazole (C) when administered as metronidazole 500 mg or ceftazidime 2000 mg-avibactam 500 mg plus metronidazole 500 mg. Linear (top) and semilog scales (bottom) are shown. AVI, avibactam; CAZ, ceftazidime; MTZ, metronidazole; SD, standard deviation. ^a^One subject did not receive the infusion of metronidazole alone as he withdrew consent prior to commencing treatment in period 3 (see main text). ^b^One subject had a metronidazole infusion 20-min longer than planned on Day 4 during the ceftazidime 2000 mg-avibactam 500 mg plus metronidazole 500 mg treatment, and the metronidazole data were excluded from the Day 4 analysis.

In agreement with the results from the CAZ-AVI PK part of the CAZ and AVI drug–drug interaction study, ceftazidime and avibactam geometric mean concentration-time profiles (Fig.2) and pharmacokinetic parameters (Table3) in the CAZ and AVI interaction part of the study were similar following administration of a single dose on Day 1 and on Day 4 (after multiple dosing [q8 h] on Days 2 and 3 and a final single dose on Day 4), with no obvious accumulation of ceftazidime or avibactam. Likewise, in the CAZ-AVI and MTZ interaction study, geometric mean concentration-time profiles (Fig.3) and pharmacokinetic parameters (Table4) for ceftazidime, avibactam, and metronidazole were similar after administration of single doses on Day 1 and after completion of the multiple dose period on Day 4; again, no obvious accumulation of ceftazidime, avibactam, or metronidazole was observed.

**Table 3:** Summary of key pharmacokinetic parameters: day 4 of the CAZ and AVI interaction part of the CAZ and AVI drug–drug interaction study

	Ceftazidime		Avibactam	
Parameter	Ceftazidime 2000 mg (*n *=* *27)	Ceftazidime 2000 mg + avibactam 500 mg (*n *=* *26)^1^	Avibactam 500 mg (*n *=* *27)	Ceftazidime 2000 mg + avibactam 500 mg (*n *=* *27)
AUC_(0-*τ*)_ (*μ*g × h/mL)	307 (18.4)	310 (20.9)	38.5 (17.9)	37.8 (18.0)
*C*_max_ (*μ*g/mL)	99.4 (16.1)	98.3 (20.9)	14.0 (16.8)	13.9 (16.3)
*t*_max_ (h)	2.00 (1.50–2.03)	2.00 (1.50–2.02)	2.00 (1.50–2.05)	2.00 (2.00–2.02)
*t*_½_ (h)	2.8 (0.1)	3.0 (0.7)	2.9 (0.5)	2.7 (0.6)

AUC, area under the plasma concentration-time curve from time zero extrapolated to infinity; AUC_(0-*τ*)_, AUC during the dosing interval; *C*_max_, maximum plasma concentration; CV, geometric coefficient of variation; SD, standard deviation; *t*_½_, terminal elimination half-life; *t*_max_, time to maximum plasma concentration. Treatments: All treatments were administered intravenously over 2 h, as a single dose on Days 1 and 4 and q8 h Days 2–3. Data for all parameters are presented as geometric mean (CV%), except for *t*_max_ which is presented as median (range) and *t*_½_ which is presented as arithmetic mean (SD).

1 PK parameters of ceftazidime from one subject receiving ceftazidime 2000 mg + avibactam 500 mg in the CAZ and AVI interaction study were excluded due to an abnormal pharmacokinetic profile for ceftazidime on Day 4.

**Table 4:** Summary of key pharmacokinetic parameters: Day 4 of the CAZ-AVI and MTZ interaction study

	Ceftazidime		Avibactam		Metronidazole	
Parameter	CAZ 2000 mg + AVI 500 mg (*n *=* *28)	CAZ 2000 mg + AVI 500 mg + MTZ 500 mg (*n *=* *28)	CAZ 2000 mg + AVI 500 mg (*n *=* *28)	CAZ 2000 mg + AVI 500 mg + MTZ 500 mg (*n *=* *28)	MTZ 500 mg (*n *=* *27)^1^	CAZ 2000 mg + AVI 500 mg + MTZ 500 mg (*n *=* *27)^1^
AUC_(0-*τ*)_ (*μ*g × h/mL)	260 (13.7)	250 (13.4)	36.5 (11.9)	37.8 (13.8)	115.0 (19.4)	121.0 (19.0)
*C*_max_ (*μ*g/mL)	78.4 (15.2)	77.3 (14.5)	13.0 (14.0)	13.2 (13.7)	21.0 (19.7)	21.4 (17.4)
*t*_max_ (h)	2.00 (1.50–2.02)	1.98 (1.48–2.03)	1.50 (1.50–2.02)	1.98 (1.48–2.03)	1.00^2^(1.00–1.50)	1.00^2^(1.00–2.50)
*t*_½_ (h)	2.7 (0.1)	2.7 (0.1)	2.6 (0.6)	2.6 (0.5)	8.4 (1.5)	9.0 (1.5)

AUC, area under the plasma concentration-time curve from time zero extrapolated to infinity; AUC_(0-*τ*)_, AUC during the dosing interval; AVI, avibactam; CAZ, ceftazidime; *C*_max_, maximum plasma concentration; CV, geometric coefficient of variation; MTZ, metronidazole; SD, standard deviation; *t*_max_, time to maximum plasma concentration; *t*_½_, terminal elimination half-life. Treatment: ceftazidime–avibactam administered intravenously over 2 h; metronidazole given intravenously over 1 h; CAZ-AVI and MTZ, MTZ administered intravenously over 1 h followed by ceftazidime–avibactam administered over 2 h. All administered as a single dose on Days 1 and 4 and q8 h on Days 2–3. Data for all parameters are presented as geometric mean (CV%), except for *t*_max_ which is presented as median (range) and *t*_½_ which is presented as arithmetic mean (SD).

1 Metronidazole parameters from one subject receiving ceftazidime–avibactam plus metronidazole were excluded due to the subject receiving a 20−min longer than planned metronidazole infusion on Day 4.

2 *n *=* *28.

In all the studies, on average, approximately 91–100% of the administered ceftazidime dose and 95–100% of the administered avibactam dose had been excreted in urine within 24 h on Day 1 and within 8 h on Day 4, consistent with previous reports (Welage et al. 1984; Vishwanathan et al. 2014).

### Assessment of drug–drug interactions

The geometric mean concentration-time profiles were similar for ceftazidime and avibactam whether administered separately or in combination in the CAZ and AVI interaction part of the CAZ and AVI drug–drug interaction study (Fig.2). Ceftazidime, avibactam, and metronidazole geometric mean concentration-time profiles were also similar whether administered separately as ceftazidime–avibactam and metronidazole, or in combination as metronidazole followed by ceftazidime–avibactam in the CAZ-AVI and MTZ interaction study (Fig.3).

**Figure 4:**
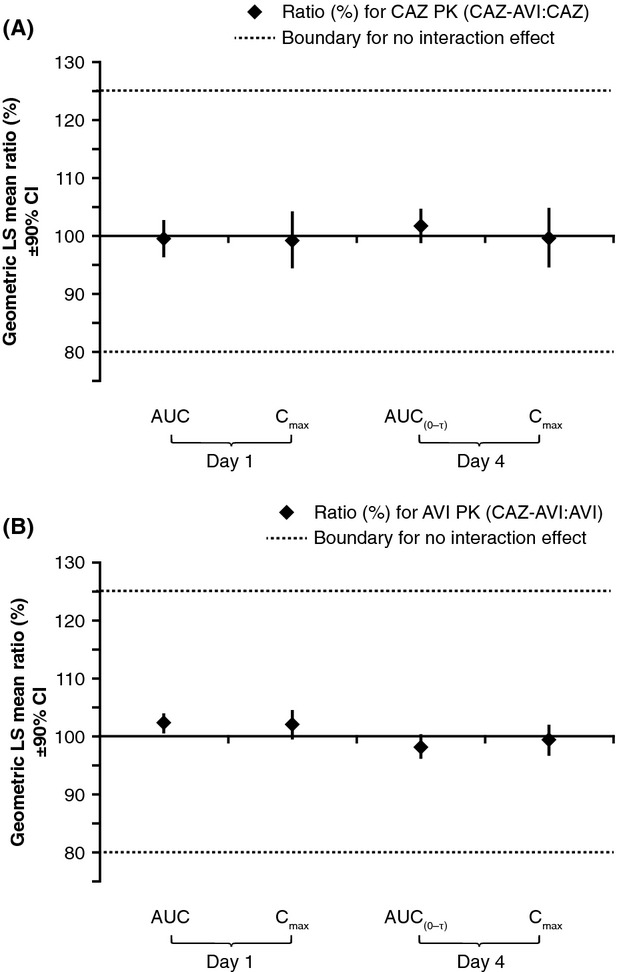
No drug–drug interaction was observed between ceftazidime and avibactam as demonstrated by the geometric LS mean (90% CI) ratios (shown as percentages) of (A) ceftazidime pharmacokinetic parameters when administered in combination as ceftazidime 2000 mg-avibactam 500 mg or separately as ceftazidime 2000 mg alone, and (B) avibactam pharmacokinetic parameters when administered in combination as ceftazidime 2000 mg-avibactam 500 mg or separately as avibactam 500 mg alone. Predefined intervals for no interaction effect are indicated by the dotted lines (data from CAZ and AVI interaction part of the CAZ and AVI drug–drug interaction study, *n* = 27). AVI, avibactam; AUC, area under the curve; AUC_(0-*τ*)_, AUC during the dosing interval; CAZ, ceftazidime; CI, confidence interval; *C*_max_, maximum plasma concentration; LS, least-squares; PK, pharmacokinetic.

**Figure 5:**
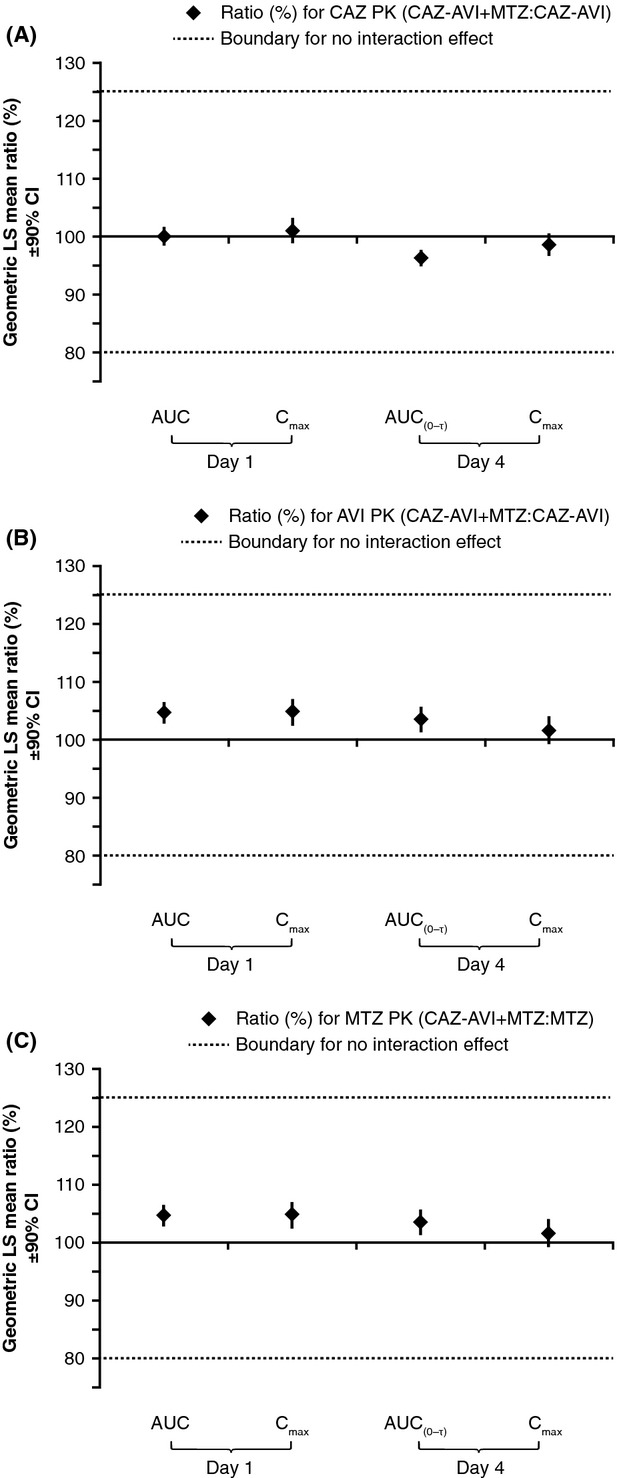
No drug–drug interaction was observed between ceftazidime–avibactam and metronidazole as demonstrated by the geometric LS mean (90% CI) ratios (shown as percentages) for pharmacokinetic parameters of (A) ceftazidime and (B) avibactam when administered as ceftazidime 2000 mg-avibactam 500 mg plus metronidazole 500 mg or ceftazidime 2000 mg-avibactam 500 mg, and (C) metronidazole when administered as ceftazidime 2000 mg-avibactam 500 mg plus metronidazole 500 mg or metronidazole 500 mg. Predefined intervals for no interaction effect are indicated by dotted line (data from CAZ-AVI and MTZ interaction study, *n *=* *28) AVI, avibactam; AUC, area under the curve; AUC_(0-*τ*)_, AUC during the dosing interval; CAZ, ceftazidime; CI, confidence interval; *C*_max_, maximum plasma concentration; LS, least-squares; MTZ, metronidazole; PK, pharmacokinetic.

The 90% CIs for the ratios of geometric LS means of the AUC and *C*_max_ of ceftazidime when administered separately or in combination with avibactam, and for avibactam when administered separately or in combination with ceftazidime, were well within the predefined interval range of 80–125% for no interaction effect on both Day 1 and Day 4 (Fig.4). Furthermore, the 90% CIs for the ratios of geometric LS means of pharmacokinetic parameters of ceftazidime and avibactam for ceftazidime–avibactam administered alone or in combination with metronidazole on Days 1 and 4 were also well within the predefined interval of 80–125% for no effect (Fig.5A and B). Similarly, 90% CIs for the ratios of geometric LS means of pharmacokinetic parameters of metronidazole when administered alone or in combination with ceftazidime–avibactam were well within the predefined interval of 80–125% for no effect (Fig.5C).

### Safety

There were no deaths, serious AEs or AEs of severe intensity in either study. In the CAZ-AVI PK part of the CAZ-AVI drug–drug interaction study, nine of 16 subjects (56.3%) had at least one AE (Table5), the most common of which were abnormal urine odor (18.8% of subjects), catheter site pain (18.8%), headache (12.5%), and back pain (12.5%). In the CAZ and AVI interaction part, 16 of 27 subjects (59.3%) had at least one AE (Table5), the most common of which were abnormal urine odor (18.5% of subjects), diarrhea (18.5%), and headache (14.8%). Most of the AEs in both parts of the study were considered by the investigator to be related to the study treatment. All AEs in the CAZ-AVI PK part of the study were considered by the investigator to be mild in intensity, with the exception of one case of back pain that was considered moderate; and all AEs in the CAZ and AVI interaction part of the study were considered to be mild, with the exception of five AEs of headache in three subjects and one AE of angioedema which were considered moderate.

**Table 5:** Number (%) of subjects with at least one AE in both parts of the CAZ and AVI drug–drug interaction study

	CAZ-AVI PK (*n *=* *16)	CAZ+AVI interaction (all groups combined)^1^(*n *=* *27)
Subjects with any AE	9 (56.3)	16 (59.3)
AEs occurring in two or more subjects overall		
Abnormal urine odor	3 (18.8)	5 (18.5)
Diarrhea	1 (6.3)	5 (18.5)
Headache	2 (12.5)	4 (14.8)
Constipation	0 (0.0)	2 (7.4)
Catheter site pain	3 (18.8)	0 (0.0)
Back pain	2 (12.5)	0 (0.0)
Pain in extremity	1 (6.3)	1 (3.7)
Syncope	0 (0.0)	2 (7.4)
Upper respiratory tract infection	1 (6.3)	1 (3.7)

AE, adverse event; AVI, avibactam; CAZ, ceftazidime; PK, pharmacokinetic. All randomized subjects were included in the safety population. AEs reported in the washout between treatments were attributed to the last treatment received.

1 Incidence of AEs was similar in each of the three treatment periods in the CAZ and AVI interaction study (AEs were reported for eight subjects (29.6%) after avibactam 500 mg, eight subjects (29.6%) after ceftazidime 2000 mg, and nine subjects (33.3%) after ceftazidime-avibactam).

Increases in serum alanine aminotransferase (ALT) of <3 × upper limit of normal (ULN) were observed in six subjects (two subjects had ALT 2.5 × ULN and 2.6 × ULN, respectively; the remaining four subjects had increases of <2 × ULN). These returned to the normal range by 12 days after the last administration of the study treatment in five of the six subjects and, in the sixth, ALT was slightly above the normal range 7 days after completing the dosing period. No elevations in aspartate aminotransferase, bilirubin, or alkaline phosphatase were observed in any subject in either part of the CAZ and AVI drug–drug interaction studies, and no clinical symptoms attributed to hepatic disorder were observed. There were no new trends or clinically significant changes in any other laboratory safety variables, vital signs or ECG in either study.

Overall, in the CAZ-AVI and MTZ interaction study, 22 of 28 subjects (78.6%) reported an AE (Table6), all of which were considered to be mild in intensity. After separate infusions of either ceftazidime–avibactam or metronidazole, the most common AE was contact dermatitis (17.9% and 7.4% of subjects, respectively), of which no cases were considered by the investigator to be related to the study treatment. After combination treatment, the most frequently reported AEs were headache (14.3% of subjects), contact dermatitis (10.7%) and diarrhea (10.7%) (Table6). Only diarrhea was considered by the investigator to be related to the study drug.

**Table 6:** Number (%) of subjects with at least one AE in the CAZ-AVI and MTZ interaction study

	CAZ-AVI (*n *=* *28)	MTZ (*n *=* *27)	CAZ-AVI and MTZ (*n *=* *28)	Overall (*n *=* *28)
Subjects with any AE	12 (42.9)	10 (37.0)	15 (53.6)	22 (78.6)
AEs occurring in two or more subjects overall				
Contact dermatitis	5 (17.9)	2 (7.4)	3 (10.7)	10 (35.7)
Headache	1 (3.6)	0 (0.0)	4 (14.3)	4 (14.3)^1^
Diarrhea	0 (0.0)	1 (3.7)	3 (10.7)	4 (14.3)
Sunburn	1 (3.6)	1 (3.7)	2 (7.1)	4 (14.3)
Vessel puncture site hemorrhage	1 (3.6)	1 (3.7)	1 (3.6)	3 (10.7)
Abdominal pain	0 (0.0)	1 (3.7)	1 (3.6)	2 (7.1)
Back pain	0 (0.0)	0 (0.0)	2 (7.1)	2 (7.1)
Catheter site hematoma	1 (3.6)	1 (3.7)	0 (0.0)	2 (7.1)
Dry mouth	1 (3.6)	1 (3.7)	0 (0.0)	2 (7.1)
Dysgeusia	0 (0.0)	0 (0.0)	2 (7.1)	2 (7.1)
Nausea	0 (0.0)	0 (0.0)	2 (7.1)	2 (7.1)
Vessel puncture site pain	0 (0.0)	1 (3.7)	1 (3.6)	2 (7.1)

AE, adverse event; AVI, avibactam; CAZ, ceftazidime; MTZ, metronidazole. All randomized subjects were included in the safety population. AEs reported in the washout period between treatments were attributed to the last treatment received.

1 One subject had a headache in the ceftazidime–avibactam treatment period and also in the CAZ-AVI and MTZ treatment, hence four subjects in total experienced headache during the study.

## Discussion

This report presents the results of two phase I studies which were designed to assess the potential for drug–drug interactions between ceftazidime and avibactam, or between ceftazidime–avibactam and metronidazole in healthy subjects. The first study, the CAZ and AVI drug–drug interaction study, was conducted in two parts. The CAZ-AVI PK part was undertaken to assess the time-dependence of the pharmacokinetic parameters for ceftazidime and avibactam after a single dose on the first day of treatment, multiple dosing (q8 h) on Days 2 to 10 and a single dose on the final day of treatment (Day 11). The study confirmed that the pharmacokinetics of ceftazidime and avibactam were similar on Day 1 after a single dose, on Day 4 after multiple dosing, and on Day 11 where a final single dose was administered following the 9 days of multiple dosing from Day 2. No obvious accumulation of either ceftazidime or avibactam was observed, and these results indicate that steady state had been reached prior to Day 4, supporting the 4-day designs of the subsequent drug–drug interaction studies. This study also provided evidence that adequate plasma concentrations of ceftazidime and avibactam were maintained throughout the duration of dosing. This is an important finding as previous studies have shown that for ceftazidime–avibactam, a joint target of serum concentrations of ceftazidime above the minimum inhibitory concentration for a given pathogen and of avibactam above the critical threshold concentration of 1 mg/L, for approximately 50% of the dosing interval are associated with efficacy (Berkhout et al. 2013; Muller et al. 2013; Li et al. 2014).

After establishing the time-independence of ceftazidime and avibactam pharmacokinetics, the subsequent CAZ and AVI interaction part of the CAZ and AVI drug–drug interaction study assessed whether ceftazidime and avibactam had an effect on each other's pharmacokinetic parameters after a single dose on the first day, 2 days’ multiple dosing (q8 h) and a single dose on the final day of treatment (Day 4). The second study, the CAZ-AVI and MTZ interaction study, assessed whether ceftazidime–avibactam and metronidazole affected each other's pharmacokinetic parameters after a single dose on the first day, 2 days’ multiple dosing (q8 h) and a single dose on the final day of treatment (Day 4). Single and multiple dosing allowed for full analysis of drug–drug interaction, and also multiple-dosing is similar to the method of administration of these drugs evaluated in two phase III studies (NCT01499290 and NCT01500239) in patients with cIAI (Mazuski et al. 2015).

In agreement with the above results, the pharmacokinetics of ceftazidime and avibactam, or ceftazidime–avibactam and metronidazole in the interaction studies, were similar after single and multiple dosing and no obvious accumulation or time-dependent pharmacokinetics were observed. Analysis of the results of the CAZ and AVI interaction study indicated that ceftazidime did not alter avibactam exposure (as measured by AUC or *C*_max_), and vice versa (Fig.4). Similarly, analysis of the results of the CAZ-AVI and MTZ interaction study indicated that metronidazole (given before ceftazidime–avibactam) did not affect exposure to either ceftazidime or avibactam, and that ceftazidime–avibactam (given after metronidazole) did not affect exposure to metronidazole (Fig.5). Therefore, no drug–drug interaction was observed between ceftazidime and avibactam, and no drug–drug interaction was observed between metronidazole and ceftazidime–avibactam. This is consistent with previous data indicating avibactam to have limited propensity for drug–drug interaction (Vishwanathan et al. 2014), an important finding as ceftazidime-avibactam is likely to be coadministered with other drugs in the clinical setting of serious infections in hospitalized patients.

Although the study protocol permitted subjects of either sex to be recruited, only male volunteers were enrolled. However, this is not anticipated to impact the study results as previous studies have not found any clinically significant differences in the pharmacokinetics of ceftazidime or avibactam between men and women (Sommers et al. 1983; Tarral and Merdjan 2015). Moreover, the pharmacokinetic results observed here are in line with previous studies investigating avibactam alone or avibactam in combination with ceftazidime (Vishwanathan et al. 2014; Merdjan et al. 2015; Tarral and Merdjan 2015).

Abnormal urine odor, diarrhea, headache, and back pain were among the most commonly reported AEs in the healthy subjects included in these studies, and there did not appear to be a trend in the reporting of any other AEs. Overall, the safety profile and tolerability of ceftazidime-avibactam did not appear to be impacted by prior administration of metronidazole. These data are in line with phase II studies in patients with cUTI, which showed the most common AEs with ceftazidime-avibactam to be headache, abdominal pain, constipation, and anxiety (Vazquez et al. 2012). In patients with cIAI the most commonly reported AEs during ceftazidime-avibactam plus metronidazole treatment were vomiting, nausea and pyrexia (Lucasti et al. 2013).

Transient elevation in serum ALT was observed in some subjects following 11 days’ single and multiple dosing with ceftazidime–avibactam in the CAZ-AVI PK part of the CAZ and AVI drug–drug interaction study; however, there were no concomitant elevations in other liver enzymes, and no clinical symptoms related to a liver disorder. Transient elevation of liver enzymes has been reported previously with ceftazidime–avibactam (Vazquez et al. 2012; Lucasti et al. 2013) and is a known effect of the cephalosporins, including ceftazidime (GlaxoSmithKline 2013). Therefore, the observation of reversible and transient ALT elevation in this part of the study is not considered to alter the risk–benefit profile of ceftazidime–avibactam and, the pharmacokinetic and safety data reported here support further investigation of ceftazidime–avibactam 2000–500 mg administered, with the addition of metronidazole 500 mg in infections where anaerobic pathogens are suspected. Moreover, the US Food and Drug Administration has recently approved the use of ceftazidime–avibactam for treatment of cIAI (in combination with metronidazole) and cUTI including acute pyelonephritis, where there are limited treatment options (Food and Drug Administration 2015).

These studies demonstrated that, in healthy subjects, the pharmacokinetic parameters of both ceftazidime and avibactam were similar after a single dose and multiple dosing, with steady-state of both drugs achieved by Day 4 and no obvious drug accumulation over 10 days of administration. Similarly, in the CAZ-AVI and MTZ interaction study, no obvious accumulation of ceftazidime, avibactam or metronidazole was observed following 2 days of multiple dosing flanked by a single dose on Day 1 and Day 4. No drug–drug interactions were demonstrated between ceftazidime and avibactam, or between metronidazole and ceftazidime–avibactam, an important finding given that these drugs will be used in combination in the clinic. Furthermore, there were no unexpected safety concerns in these studies, and the safety profile and tolerability of ceftazidime–avibactam was not affected by coadministration of metronidazole. These results support the dosing regimen used in phase III clinical trials in patients with cIAI (NCT01726023, NCT01499290, and NCT01500239), cUTI (NCT01595438 and NCT01599806), and nosocomial pneumonia (NCT01808092).

## Conclusions

Pharmacokinetic and safety profiles of ceftazidime and avibactam were similar when given alone or in combination and were not affected by the addition of metronidazole. There was no drug–drug interaction between ceftazidime and avibactam, and ceftazidime–avibactam and metronidazole. This supports the dosing regimen being evaluated in ceftazidime–avibactam phase III trials.

## Abbreviations

AE: adverse events
AUC: area under curve
C_max_: maximum plasma concentration
C_last_: last quantifiable plasma concentration
CL_R_: renal clearance
ALT: alanine aminotransferase
cIAI: complicated intra-abdominal infections
CI: confidence intervals
cUTI: complicated urinary tract infections
CV: coefficient of variation
ECG: electrocardiogram
IV: intravenous
LLOQ: lower limit of quantification
LS: least-squares
RE: relative error
SD: standard deviation
ULN: upper limit of normal
